# T cell deficiency in spinal cord injury: altered locomotor recovery and whole-genome transcriptional analysis

**DOI:** 10.1186/s12868-015-0212-0

**Published:** 2015-11-06

**Authors:** David Satzer, Catherine Miller, Jacob Maxon, Joseph Voth, Christina DiBartolomeo, Rebecca Mahoney, James R. Dutton, Walter C. Low, Ann M. Parr

**Affiliations:** Department of Neurosurgery, University of Minnesota, D429 Mayo Memorial Building, MMC 96, 420 Delaware Street, SE, Minneapolis, MN 55455 USA; Stem Cell Institute, University of Minnesota, Minneapolis, MN 55455 USA

**Keywords:** Spinal cord injury, Inflammation, Locomotor function, Neuronal cell death, Axonal regeneration

## Abstract

**Background:**

T cells undergo autoimmunization following spinal cord injury (SCI) and play both protective and destructive roles during the recovery process. T cell-deficient athymic nude (AN) rats exhibit improved functional recovery when compared to immunocompetent Sprague–Dawley (SD) rats following spinal cord transection.

**Methods:**

In the present study, we evaluated locomotor recovery in SD and AN rats following moderate spinal cord contusion. To explain variable locomotor outcome, we assessed whole-genome expression using RNA sequencing, in the acute (1 week post-injury) and chronic (8 weeks post-injury) phases of recovery.

**Results:**

Athymic nude rats demonstrated greater locomotor function than SD rats only at 1 week post-injury, coinciding with peak T cell infiltration in immunocompetent rats. Genetic markers for T cells and helper T cells were acutely enriched in SD rats, while AN rats expressed genes for T_h_2 cells, cytotoxic T cells, NK cells, mast cells, IL-1a, and IL-6 at higher levels. Acute enrichment of cell death-related genes suggested that SD rats undergo secondary tissue damage from T cells. Additionally, SD rats exhibited increased acute expression of voltage-gated potassium (K_v_) channel-related genes. However, AN rats demonstrated greater chronic expression of cell death-associated genes and less expression of axon-related genes. Immunostaining for macrophage markers revealed no T cell-dependent difference in the acute macrophage infiltrate.

**Conclusions:**

We put forth a model in which T cells facilitate early tissue damage, demyelination, and K_v_ channel dysregulation in SD rats following contusion SCI. However, compensatory features of the immune response in AN rats cause delayed tissue death and limit long-term recovery. T cell inhibition combined with other neuroprotective treatment may thus be a promising therapeutic avenue.

**Electronic supplementary material:**

The online version of this article (doi:10.1186/s12868-015-0212-0) contains supplementary material, which is available to authorized users.

## Background

The role of the immune system in spinal cord injury (SCI) is complex, controversial, and relevant to the pathophysiology and treatment of SCI. The literature supports both positive and negative effects of the immune system in functional recovery after SCI [1, 2]. T cells, which help coordinate the immune response, have received substantial attention for their role in SCI.

Systemic spread of central nervous system (CNS) antigens after SCI leads to the formation of autoimmune T cells [3, 4] that migrate to the injury site, reaching peak accumulation at 1 week post-injury [5, 6]. Helper T cells are responsible for macrophage recruitment and activation, which has been shown to produce focal axonal injury and demyelination [7, 8]. These cells also cause formation of autoimmune B cells that produce antibodies to CNS proteins [9]. Meanwhile, autoimmune cytotoxic T cells have been demonstrated to produce significant white matter pathology [10, 11]. Interestingly, while myelin-reactive T cells are associated with tissue loss and functional impairment [12, 13], these same cells have been found to secrete neuroprotective factors and prevent secondary damage to the spinal cord via “protective autoimmunity” [14–16].

T cell suppression appears to improve recovery from SCI. T cell inhibitors including cyclosporine [17, 18], fingolimod [19], and integrin inhibitors [20, 21] have been shown to improve neurological function after SCI, though their benefit may depend upon injury severity [22]. T cell-deficient athymic nude (AN) rats demonstrate superior locomotor function after transection injury compared to immunocompetent Sprague–Dawley (SD) rats [23]. The effects of T cell deficiency in nervous system trauma are not limited to SCI; T and B cell-deficient *RAG1*^−/−^ mice suffer less damage from traumatic brain injury [24].

In order to characterize the whole-genome response to SCI, researchers have employed microarrays [25, 26] and RNA sequencing (RNA-seq) [27]. Gene transcripts associated with inflammation, cell death and survival, tissue and vascular repair, and nervous system function and development are most highly represented in the injury epicenter [25–29]. The only study to examine gene expression differences between AN and SD rats [25] employed microarray analysis—a more limited technique than RNA-seq—and found only 80 genes that differed between the two strains.

In the present study, we assessed (1) locomotor recovery and (2) gene expression in the injury epicenter, following SCI, in both SD and AN rats. We focused on the acute (1 week post-injury, when the T cell response is maximal) and chronic (8 weeks post-injury) phases of recovery. Based on previously findings after spinal cord transection, we hypothesized that AN rats would show sustained superior locomotor function after injury compared to SD rats. We subsequently used RNA-seq to identify gene expression differences between the two strains that could explain variation in locomotor function.

## Methods

### Spinal cord injury and postoperative care

All studies were approved by the University of Minnesota Institutional Animal Care and Use Committee (IACUC). Age-matched adult (16–18 week old) female SD (Hsd:SD, N = 15) and AN (Hsd:RH-*rnu*/*rnu*, N = 15) rats (Charles River, Wilmington, MA, USA) received moderate contusion injury using the Infinite Horizon impactor (Precision Systems, Lexington, KY, USA). Briefly, animals were anesthetized with isoflurane and underwent T8/T9 laminectomy followed by spinal cord impact at a force of 200 kdyn. Animals received buprenorphine (0.05 mg/kg SC q12 h) for 3 days after surgery to moderate acute pain, and amoxicillin (14 g/L in drinking water) for 7 days after surgery to prevent urinary tract infection as part of standard postoperative care. Urine was expressed manually twice daily for the first 3 days after surgery and daily thereafter until the return of independent urination. Animals were assigned to either the acute (1 week, N = 5 per strain) or chronic (8 weeks, N = 10 per strain) recovery phase groups.

### Functional assessment

Locomotion was assessed by two observers using the Basso, Beattie, and Bresnahan (BBB) scale [30] on the second postoperative day and at the end of each week following surgery. Animals were on site for at least 1 week prior to SCI to acclimate to the environment and handling by the observers. Raters were trained by a researcher (A.P.) with extensive experience using the BBB scale. BBB scores were compared between strains via repeated-measures analysis of variance.

### Tissue preparation

At 1 or 8 weeks after injury, animals were anesthetized with ketamine (100 mg/kg IP) and xylazine (10 mg/kg IP). For RNA-seq analysis, animals underwent transcardial perfusion with phosphate buffered saline (PBS), and a 1-mm spinal cord segment through the injury site was harvested and flash-frozen in liquid nitrogen. For histological analysis, animals underwent transcardial perfusion with 4 % paraformaldehyde. The spinal cord was removed and cryoprotected in 30 % sucrose.

### Histology

Spinal cord segments from some animals sacrificed 1 week post-injury (acute recovery group) were frozen in OCT compound and sectioned in the parasagittal plane at a thickness of 10 μm. Representative sections for each spinal cord were selected from the mid-sagittal area and immunostained. The following antibodies were used: anti-CD68 (1:500, AbD Serotec, Raleigh, NC, USA) for macrophages, anti-CD16 (1:100, Abcam, Cambridge, United Kingdom) for M1 macrophages, anti-CD163 (1:50, AbD Serotec) for M2 macrophages, donkey anti-rabbit IgG (1:1000, ThermoFisher Scientific, Carlsbad, CA, USA), and donkey anti-mouse IgG (1:1000, Abcam). Negative controls were obtained by omission of the primary antibody. Immunofluorescent photomicrographs were taken from a 1-mm segment centered on the lesion epicenter.

Immunofluorescence images were analyzed using ImageJ (National Institutes of Health, Bethesda, MD, USA). Threshold values for excluding background fluorescence were determined as the 95th percentile of pixel intensity averaged across all negative control images for each immunostain. The Analyze Particle function was then used to determine the percent of tissue area stained. Percent area was compared between AN and SD groups via the two-tailed Student’s t test using an alpha value of 0.05.

### RNA extraction and sequencing

RNA extraction, cDNA library creation, and RNA-seq were performed with the assistance of the University of Minnesota Biomedical Genomics Center staff. RNA was extracted from tissue samples using the RNeasy Mini Kit (Qiagen, Venlo, The Netherlands). RNA quantity and quality were assessed with the Aligent 2100 Bioanalyzer (Aligent Technologies, Santa Clara, CA, USA). The polyadenylated coding mRNA in each extract (N = 3 for each strain–time point combination) was isolated and reverse transcribed using random primers. The resulting paired-end cDNA libraries were subsequently sequenced using the HiSeq 2000 (Illumina, San Diego, CA, USA). For each sample, at least 40 million paired-end reads of 51 base pairs were performed.

### Read mapping and transcript identification

Analysis of RNA-seq data was carried out using the Galaxy software platform [31–33] hosted by the Minnesota Supercomputing Institute. Reads first underwent quality control assessment with the FastQC tool and adapter sequence contamination removal with the CutAdapt tool. Because all base positions had first-quartile Phred scores greater than 20, trimming with FastQ Trimmer was not needed. Reads were subsequently mapped to the *Rattus norvegicus* RN4 reference genome with TopHat (version 1.5.0) [34] using an empirically determined insertion size of 210 base pairs. The mapped reads were assembled into transcripts with Cufflinks (version 0.0.6) [35] using quartile normalization. Transcriptional datasets for each time point were pooled using CuffMerge, and differences between strains at each time point were identified with CuffDiff. Gene expression differences with a Q value (false discovery rate-adjusted P value) less than 0.05 were considered to be statistically significant.

### Immune and neural marker genes

To measure the presence and activity of both immune and neural cells, we first identified a variety of genetic markers for different cell types belonging to the innate immune system, adaptive immune system, and CNS as follows: dendritic cell (*CD83*), macrophage (*ITGAM*, *CD68*, *CD14*), M1 macrophage (*CD86*, *RBPJ*), M2 macrophage (*ARG1*, *CMAF*), mast cell (*FCER1A*, *ENPP3*, *KIT*), granulocyte (*CEACAM3*, *LY6C*), NK cell (*NCR3*, *KLRA1*, *KLRA2*), T cell (*CD3d*, *CD3e*, *CD3g*), helper T cell (*CD4*), T_h_1 cell (*IL12Rb1*, *CXCR3*, *CCR5*), T_h_2 cell (*IL1R1*, *IL2R2*, *IL2RL1, CXCR4, CCR3, CCR4, CCR7, CCR8, TNFRSF8*), cytotoxic T cell (*CD8a, CD8b, GZMB, TIA1*), B cell (*CD19, CD20*), neuron (*NEFH, RBFOX3*), oligodendrocyte (*APC, MBP, PLP*), and astrocyte (*GFAP*). For each cell type, the marker with the greatest abundance (fragments per kilobase of exon per million fragments mapped, FPKM) summed over the four conditions (strain–time point combinations) was used as the final cell marker.

### Broad patterns of differential gene expression

In order to broadly understand strain-dependent differences in biological processes in response to injury, we categorized a subgroup of genes using gene set enrichment analysis (GSEA). Specifically, genes differentially expressed between strains in the acute phase only were examined via GSEA. First, rat genome database (RGD) gene identifiers were converted to gene symbols using the DAVID Gene ID Conversion Tool (http://david.abcc.ncifcrf.gov). Gene symbols for differentially expressed genes were then entered into the GSEA utility (http://www.broadinstitute.org/gsea) and mapped to the Canonical Pathways gene set collection. The 20 most highly enriched gene sets were identified.

### Investigation of specific pathophysiological processes

To investigate differences in specific processes (e.g. cell death) between strains, we identified appropriate gene ontology (GO) terms related to those processes (refer to “Results” section for a list of all GO terms used). Along with these GO terms, we entered genes differentially expressed in the acute and/or chronic phases into the AmiGO Slimmer tool (http://amigo1.geneontology.org). This allowed us to compare gene expression in processes of interest between strains at different time points.

## Results

### Locomotor recovery

**Fig. 1:**
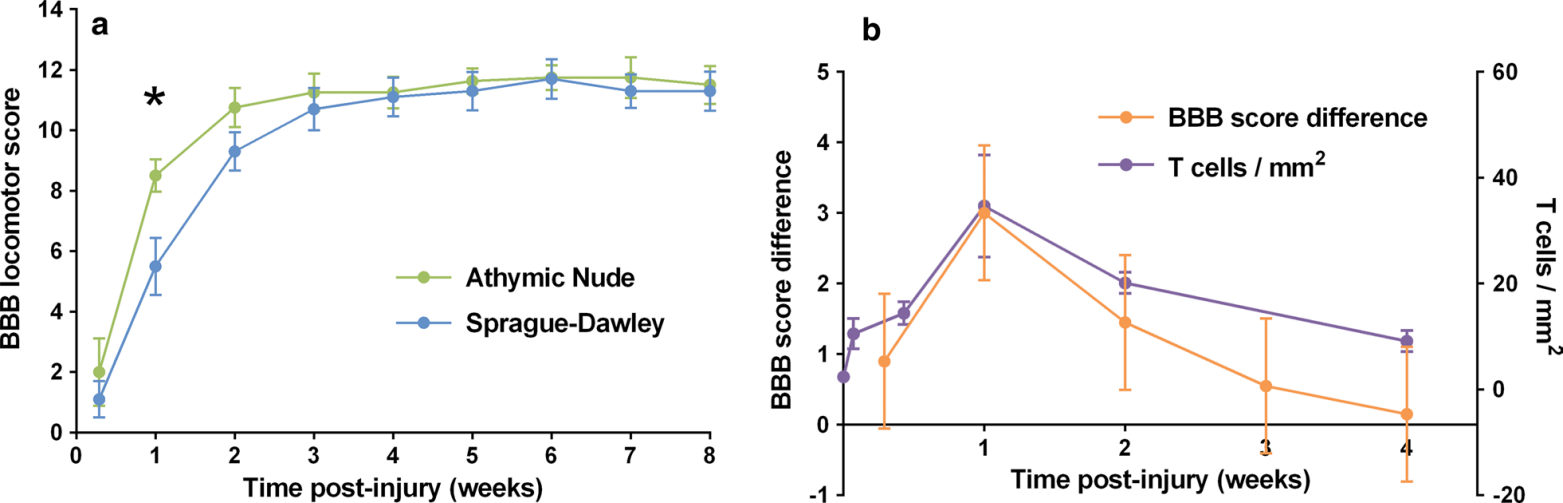
Variation in locomotor recovery between AN and SD rats. **a** Mean BBB locomotor score over time following SCI. *Asterisk* indicates P < 0.05. N = 8 for AN rats; N = 10 for SD rats. *Error bars* represent ±1 standard error. **b** Locomotor difference and T cell infiltration. Mean BBB score difference between AN and SD rats (*left axis*) and T cell density (T cells/mm^2^) in injury epicenter of SD rat (*right axis*). T cell data is used with permission [5]. *Error bars* represent ±1 standard error

A total of 15 AN and 15 SD rats underwent contusive SCI at a force of 200 kdyn. The BBB locomotor scores of AN and SD rats differed (P = 0.02) at 1 week following injury, at which point the mean BBB scores (±1 standard error) were 8.5 ± 0.5 and 5.5 ± 0.9, respectively (Fig. 1a). The BBB scores of the two strains did not differ at any other time point.

Figure 1b shows the time course of the difference between BBB scores of AN and SD rats as well as the density of the T cell infiltrate in the SD rat injury epicenter (data used with permission) [5] over the first 4 weeks after injury. The locomotor advantage of AN rats over SD rats at 1 week coincides with the maximal T cell infiltrate, and both functional difference and T cell density decrease gradually thereafter.

### RNA-seq experimental design

Based upon the locomotor difference between AN and SD rats in the acute (1 week post-injury) but not chronic (8 weeks post-injury) phase of recovery, we took a multi-step approach to analysis of RNA-seq data. First, to gain a broad understanding of the transcriptional basis of the acute locomotor difference, we compared the acute- and chronic-phase differential expression profiles to identify genes that were differentially expressed in the acute phase only. We then identified physiological pathways that were highly represented among these genes. Second, to investigate specific pathophysiological processes involved in SCI, we identified genes that were differentially expressed—in the acute and/or chronic phase—and associated with select GO terms.

### Public data availability

RNA-seq data (raw and processed files) are available in the Gene Expression Omnibus (http://www.ncbi.nlm.nih.gov/geo) under accession number GSE62760.

### Quality control

**Fig. 2:**
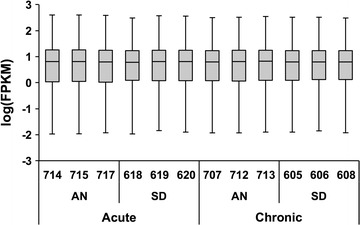
Expression ranges for individual tissue samples. *Box*-and-*whisker* plot of log(FPKM) for each animal (3-digit identification number). *Whiskers* indicate 1st to 99th percentile; values outside this range are not represented. Note that the expression ranges were highly consistent between samples

RNA samples sent for sequencing had 1.8–10 ng of RNA at a concentration of 92–100 ng/µL and an RNA integrity number (RIN) of 9.7–10. For each sample, 40.0–51.6 million reads 51 base pairs in length were sequenced. Both paired read sets for every sample had a per-base first-quartile Phred quality score greater than 30 for all bases, indicating a base measurement error less than 0.1 %. Expression ranges were highly consistent between samples (Fig. 2).

### Differential gene expression

**Fig. 3:**
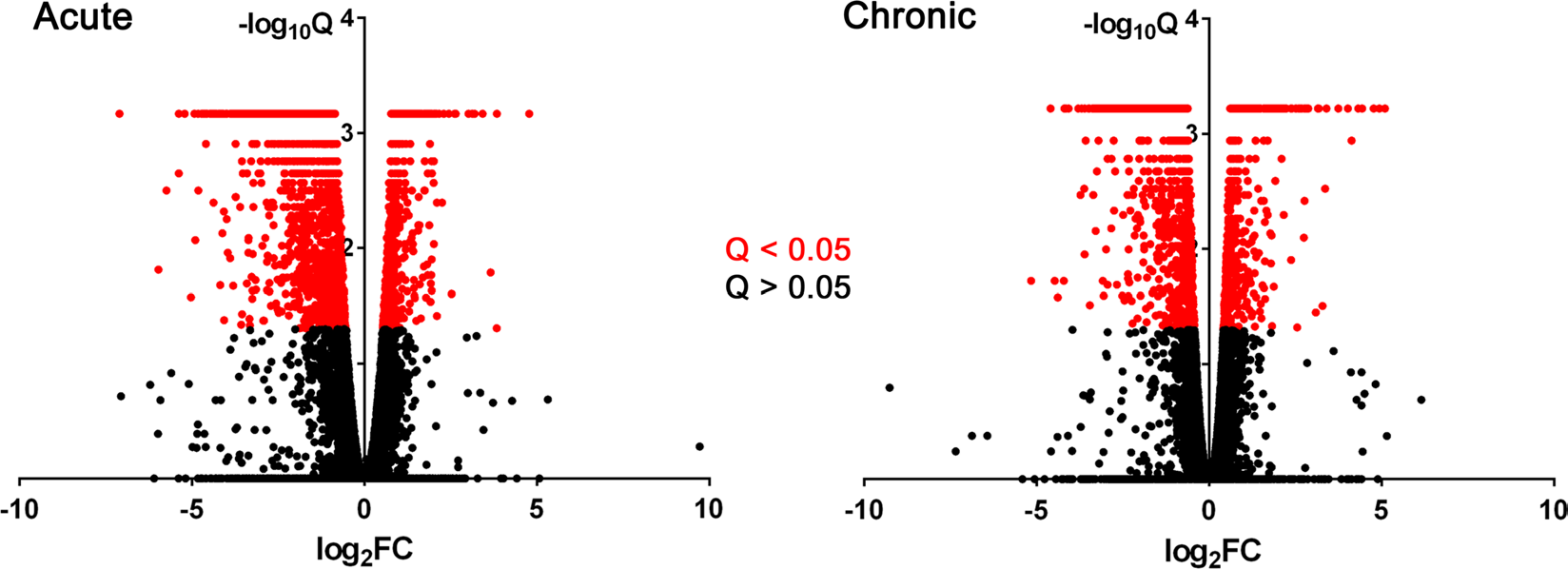
Volcano plots for differential gene expression in the acute and chronic phases of SCI. *Colors* represent Q < 0.05 (*red*) and Q > 0.05 (*black*) for differential expression testing. The overall magnitude of differential gene expression was greater in the acute phase, as evidenced by a larger median absolute value of log_2_FC (1.27 acute versus 0.99 chronic; P < 0.001) for differentially expressed genes. *FC* fold change (AN FKPM divided by SD FKPM). Q, false discovery rate-adjusted P value

A total of 14,911 genes were identified by RNA-seq: 14,565 in the acute phase and 14,567 in the chronic phase. Read mapping statistics for each sample are summarized in Table 1. Statistical significance for differential gene expression was determined based upon the Q value (false discovery rate-adjusted P value). Magnitude of differential expression was quantified as fold change (FC), equal to AN FKPM divided by SD FKPM. Volcano plots of statistical significance (−log_10_Q) versus difference magnitude (log_2_FC) reveal that the magnitude of differential gene expression was larger in the acute phase (Fig. 3). The median absolute value of log_2_FC for differentially expressed genes was 1.27 in the acute phase, compared to 0.99 in the chronic phase (P < 0.001 for Mann–Whitney U test).

**Table 1:** Read mapping from RNA-seq output

Phase	Strain	Animal	Total reads	Mapped reads	% Mapped	Genes
Acute	AN	714	51,634,511	50,136,688	97.1	13,574
		715	44,269,036	43,001,258	97.1	13,471
		717	44,842,419	43,542,173	97.1	13,559
	SD	618	48,194,423	46,090,448	95.6	14,035
		619	39,885,927	38,511,046	96.6	13,610
		620	45,447,598	43,769,395	96.3	13,745
Chronic	AN	707	44,446,497	42,956,572	96.6	13,763
		712	45,858,389	44,358,919	96.7	13,653
		713	43,186,929	41,819,751	96.8	13,632
	SD	605	49,095,638	47,230,803	96.2	13,861
		606	45,671,290	43,873,662	96.1	13,849
		608	45,194,438	43,242,894	95.7	13,926

**Fig. 4:**
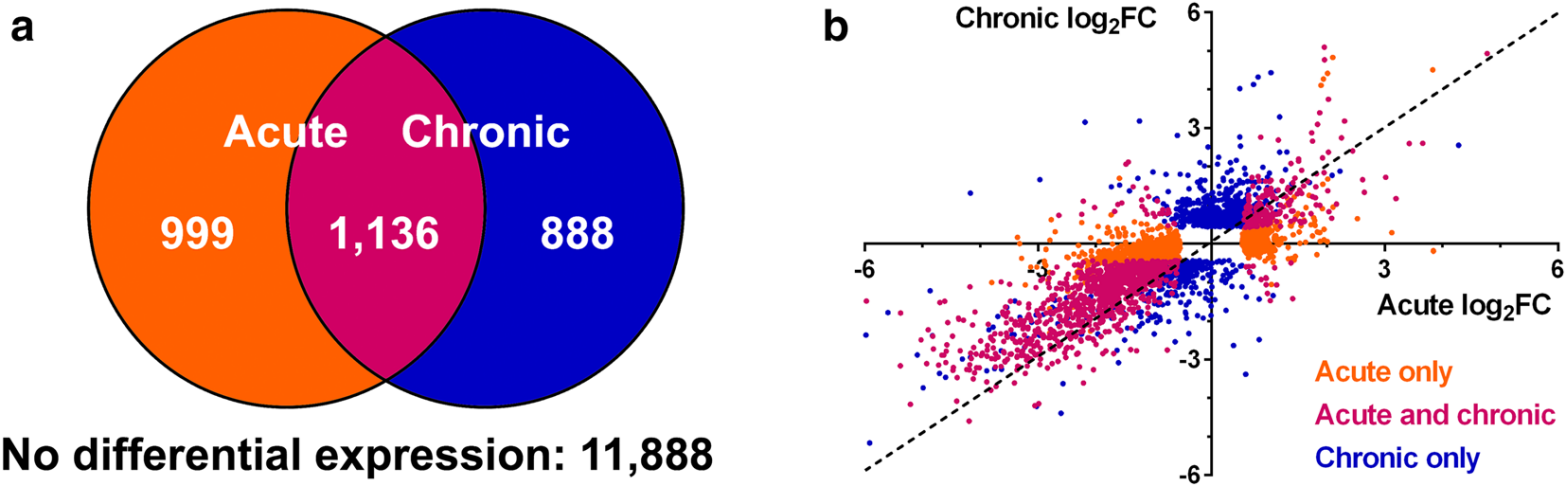
Differential gene expression in the acute and chronic phases of SCI. **a** *Venn diagram* depicting number of differentially expressed genes over time. **b** Plot of gene log_2_FC values in the acute (*horizontal axis*) and chronic (*vertical axis*) phases. *Colors* represent the phase in which genes are differentially expressed. The *dashed line* (y = x) has a slope of 1. *FC* fold change (AN FKPM divided by SD FKPM)

Of the 14,911 genes identified by RNA-seq, 11,888 displayed no differential expression (Fig. 4a). Among differentially expressed genes, 999 differed in the acute phase only, 888 in the chronic phase only, and 1136 in both acute and chronic phases. Most genes that were differentially expressed in both acute and chronic phases were greater expressed in SD rats in both phases, indicated by log_2_FC values less than zero (Fig. 4b). Forty-two genes were greater expressed in SD rats in one phase and AN rats in the other. A complete list of the identified genes, expression data, and results of significance testing is provided in Additional file 1: Table S1 (as well as in the Gene Expression Omnibus; see above).

### Immune and neural marker genes

The highest-abundance immune and neural cell marker genes were identified and compared between strains in the acute and chronic phases (Table 2). Genes corresponding to T cells (*CD3g*) and helper T cells (*CD4*) were enriched in SD rats (relative to AN rats) in the acute phase only. In AN rats, T_h_1 cell marker *CXCR4* was enriched in AN rats in both the acute and chronic phases, while cytotoxic T cell marker *CD8a* was enriched in the acute phase only. Gene markers for NK cells (*KLRA1*) and mast cells (*KIT*) were enriched in AN rats in the chronic phase only. Expression of markers for dendritic cells (*CD83*), macrophages (all: *CD68*; M1: *CD86*; M2: *CMAF*), granulocytes (*Ly6C*), T_h_1 cells (*CCR5*), and B cells (*CD19*) did not vary by strain. The neurofilament gene *NEFH* was enriched in AN rats in the chronic phase; oligodendrocyte (*PLP*) and astrocyte (*GFAP*) marker genes were not differentially expressed.

**Table 2:** Analysis of cell types via marker genes

System	Cell type	Marker	Acute			Chronic		
			Greater	log_2_FC	Q	Greater	log_2_FC	Q
Innate immune system	Dendritic cell	*CD83*		0.14	0.77		−0.02	0.98
	Macrophage	*CD68*		0.66	0.12		0.47	0.07
	M1 macrophage	*CD86*		0.50	0.16		−0.18	0.68
	M2 macrophage	*CMAF*		0.46	0.12		0.32	0.18
	Mast cell	*KIT*		−0.05	0.95	AN	0.57	0.01
	Granulocyte	*Ly6C*		0.19	0.91		0.26	0.87
	NK cell	*KLRA1*		0.63	0.15	AN	1.20	<0.01
Adaptive immune system	T cell	*CD3g*	SD	−2.37	0.02		−0.88	0.17
	Helper T cell	*CD4*	SD	−1.20	<0.01		−0.25	0.36
	T_h_1 cell	*CCR5*		−0.12	0.83		−0.14	0.72
	T_h_2 cell	*CXCR4*	AN	0.65	0.02	AN	0.49	0.01
	Cytotoxic T cell	*CD8a*	AN	1.39	<0.01		−0.37	0.16
	B cell	*CD19*		−1.29	1.00		0.74	1.00
Central nervous system	Neuron	*NEFH*		−3.31	0.05	SD	−1.90	<0.01
	Oligodendrocyte	*PLP*		−1.27	0.30		−0.21	0.82
	Astrocyte	*GFAP*		−1.48	0.34		0.00	1.00

*FC* fold change (AN FKPM divided by SD FKPM); Q, false discovery rate-adjusted P value

### Gene set enrichment analysis

The 20 most highly enriched gene sets were identified for genes differentially expressed in the acute phase only (Table 3). We then manually classified these gene sets into five themes: immune system (7 sets), neurons and axons (5 sets), GPCR signaling (3 sets), cell transport (3 sets), and other (2 sets). For ease of interpretation, percentage of genes with FC >1 in the acute period—meaning genes acutely enriched in AN rats—is reported below for all gene sets.

**Table 3:** Gene set enrichment analysis (GSEA) of genes differentially expressed in the acute phase only

Theme^a^	Gene set	Genes	% Acutely enriched		−log(Q)^b^
			AN (FC >1)	SD (FC <1)	
Immune system	Cell adhesion molecules	25	32	68	Infinite
	Cell surface interactions at the vascular wall	17	71	29	10.4
	Hematopoietic cell lineage	14	64	36	7.6
	Hemostasis	45	67	33	Infinite
	Immune system	51	76	24	10.2
	Leukocyte trans-endothelial migration	17	59	41	8.7
	Platelet activation, signaling, and aggregation	21	62	38	8.1
Neurons and axons	Axon guidance (Kegg gene set)	17	12	88	8.1
	Axon guidance (Reactome gene set)	29	17	83	12.8
	Neuroactive ligand-receptor interaction	23	22	78	7.6
	Neuronal system	32	16	84	Infinite
	Transmission across chemical synapses	24	17	83	11.5
GPCR signaling	Class A1 rhodopsin-like receptors	24	46	54	7.3
	GPCR ligand binding	32	41	59	9.9
	GPCR signaling	45	42	58	7.6
Cell transport	Solute carrier-mediated transmembrane transport	28	36	64	12.4
	Transmembrane transport of small molecules	44	32	68	Infinite
	Transport of inorganic cations/anions and amino acids/oligopeptides	19	32	68	12.4
Other	Cell cycle	17	94	6	8.2
	Developmental biology	32	22	78	10.2

*FC* fold change (AN FKPM divided by SD FKPM), *Q* false discovery rate-adjusted P value

^a^The 20 most highly enriched gene sets have been manually grouped for conceptual purposes

^b^The value of −log(Q) is given for the GSEA Q value for each gene set

Most genes belonging to immune system gene sets were greater expressed in AN rats (59–71 % with acute FC >1), with the exception of cell adhesion molecules (32 % with acute FC >1). Examination of genes belonging to the “immune system” gene set revealed that genes for cytokines IL-1a (acute FC = 2.24, Q = 0.01; chronic FC = 1.05, Q = 0.95) and IL-6 (acute FC = 3.86, Q = 0.01; chronic FC = 1.29, Q = 1.00) were among the most AN-enriched immune system-related genes in the acute phase. Genes belonging to neurons and axon gene sets were greater expressed in SD rats (12–22 % with acute FC >1). Genes belonging to GPCR signaling and cell transport gene sets were more frequently enriched in SD rats (41–46 % and 32–36 % with acute FC >1, respectively). The “other” theme consisted of the “cell cycle” and “developmental biology” gene sets. Cell cycle genes were nearly all enriched in AN rats (94 % with acute FC >1), while developmental biology genes were predominantly enriched in SD rats (22 % with acute FC >1).

### Cell death-associated genes

We identified cell death-associated genes differentially expressed in the acute and/or chronic phases (Table 4). Genes associated with the cell death parent GO term were acutely enriched in SD rats (39 % with acute FC >1) and chronically enriched in AN rats (57 % with chronic FC >1). Programmed cell death (202 genes in acute phase and 179 genes in chronic phase) was more highly represented than necrotic cell death (7 genes in acute phase and 6 genes in chronic phase). Of note, however, necrosis-associated genes were enriched in AN rats in both the acute (86 % with acute FC >1) and chronic (83 % with chronic FC >1) phases. Genes associated with regulation of cell death were acutely enriched in SD rats (39 % with acute FC >1) and chronically enriched in AN rats (58 % with chronic FC >1). Neuron death-associated genes were enriched in SD rats in both acute (21 % with acute FC >1) and chronic (41 % with chronic FC >1) phases, though less so in the chronic phase. For cell and neuron death at a given time point, the strain with greater expression of death-associated genes also had greater expression of both positive and negative death regulation-associated genes.

**Table 4:** Differential expression within gene ontologies of interest

Identifier	Gene ontology	Acute			Chronic		
		Genes	% Enriched		Genes	% Enriched	
			AN (FC >1)	SD (FC <1)		AN (FC >1)	SD (FC <1)
GO:0008219	Cell death	221	39	61	196	57	43
	Type of cell death						
GO:0012501	Programmed	202	39	61	179	56	44
GO:0070265	Necrotic	7	86	14	6	83	17
GO:0010941	Regulation of cell death	181	39	61	151	58	42
GO:0010942	Positive regulation	60	42	58	49	65	35
GO:0060548	Negative regulation	100	38	62	86	53	47
GO:0070997	Neuron death	47	21	79	37	41	59
GO:1901216	Positive regulation	11	9	91	5	40	60
GO:1901215	Negative regulation	28	21	79	26	42	58
	Voltage-gated potassium channel						
GO:0005249	Activity	27	4	96	23	4	96
GO:0008076	Complex	22	5	95	15	7	93
GO:0045163	Clustering	1	0	100	0	n/a	n/a

*FC* fold change (AN FKPM divided by SD FKPM)

### Voltage-gated potassium channel-related genes

We identified differentially expressed genes associated with voltage-gated potassium (K_v_) channels (Table 4). Genes related to K_v_ channel activity were enriched in SD rats in both the acute (4 % with acute FC >1) and chronic (4 % with chronic FC >1) phases. Similarly, genes related to the K_v_ channel complex were enriched in SD rats in both the acute (5 % with acute FC >1) and chronic (7 % with chronic FC >1) phases. One gene (*CNTN2*) related to K_v_ channel clustering was differentially expressed. *CNTN2* was differentially expressed in the acute phase (acute FC = 0.44, Q < 0.01) but not in the chronic phase (chronic FC = 0.98, Q = 0.97).

### Histologic assessment of acute macrophage infiltrate

**Fig. 5:**
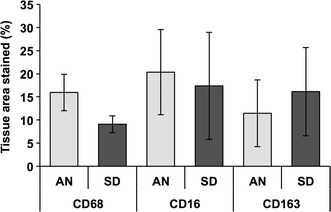
Histologic assessment of macrophage infiltrate at the acute time point (1 week post-injury). The percentage of tissue area positively stained for macrophages (CD68), M1 macrophages (CD16), or M2 macrophages (CD163) was compared between AN and SD rats (N = 4 rats per strain). No significant differences were observed (P = 0.19 for CD68, P = 0.85 for CD16, and P = 0.71 for CD163). *Error bars* represent ±1 standard error

To corroborate our gene expression data, we stained spinal cord tissue obtained 1 week post-injury for macrophages (anti-CD68) as well as the M1 (anti-CD16) and M2 (anti-CD163) macrophage subtypes. Figure 5 depicts the results of fluorescence immunostaining. Percentage of tissue area stained did not vary by strain for any macrophage marker (P = 0.19 for CD68, P = 0.85 for CD16, and P = 0.71 for CD163).

## Discussion

In this study, we found that T cell-deficient AN rats possess greater locomotor function than immunocompetent SD rats at 1 week post-SCI, but not at other time points. We examined differential gene expression between AN and SD rats using RNA-seq to clarify the physiological and cellular basis for this functional difference. A prior study of gene expression in AN and SD rats after SCI found only 80 genes whose expression differed by strain [25]. In contrast, we identified 3023 differentially expressed genes in the acute (1 week post-injury) and/or chronic (8 weeks post-injury) phases of SCI. The only published report of functional recovery after SCI in AN and SD rats found sustained locomotor advantage in AN rats following spinal cord transection [23]. We too observed functional superiority in AN rats after contusion SCI; however, this advantage was only seen at 1 week post-injury.

### Effect of T cell deficiency on locomotor recovery

Athymic Nude rats have previously been found to achieve greater locomotor function than SD rats after spinal cord transection [23]. Whereas SD rats only recovered slight movement of hindlimb joints, AN rats on average regained extensive movement in one or more hindlimb joints. Based upon this finding, we initially hypothesized that AN rats would demonstrate superior locomotor function over the entire recovery period following moderate spinal cord contusion. In our study, AN rats achieved greater BBB locomotor scores (8.5 ± 0.5) than SD rats (5.5 ± 0.9) at 1 week post-injury, with weight-supported stepping observed in some AN rats but no SD rats. However, this functional advantage was transient. The BBB score difference followed a similar time course to the magnitude of the T cell infiltration in immunocompetent SD rats [5]. Also in line with difference in functional outcome, greater magnitude of differential gene expression was observed in the acute phase of recovery than in the chronic phase.

Discrepancy between our results and those of the aforementioned study are likely due to substantial differences in the mechanism and severity of injury. The authors of the prior study conceded that axonal regrowth through the transection site was implausible and attributed prolonged functional difference to sparing of local neuronal connections and central pattern generators [23]. In contusion injury, longitudinal white matter tracts are partially spared [25, 27] and undergo some long-distance regeneration in addition to local sprouting [36, 37]. Variation in the extent and function of surviving tissue may help to account for the difference in locomotor recovery between AN and SD rats.

### Mechanistic basis of altered locomotor recovery

The pathophysiology of SCI is complex, and the processes of secondary injury, wound healing, and functional recovery are multifactorial [38]. Consequently, comprehensive analysis of these processes is difficult, despite the use of broad-spectrum techniques such as RNA-seq. We have therefore focused on specific, known pathophysiological aspects of SCI that may plausibly be related to T cell function.

The differential recovery course between SD and AN rats in this study has two possible explanations which are not mutually exclusive. One hypothesis is that while AN rats recover function more quickly after SCI, they are limited in their ultimate recovery in the same ways as SD rats. For example, while the absence of T cells may contribute to tissue sparing in the acute phase of SCI, that tissue may be gradually lost nonetheless. A second hypothesis is that AN rats are less vulnerable to temporary causes of paralysis after SCI, but functional differences disappear once those effects have subsided. These causes may include edema, metabolic dysfunction, and K_v_ channel dysregulation [38].

In our analysis, we compared components of the immune response between AN and SD rats. We then investigated strain-dependent differences in cell death, tissue repair, and K_v_ channel function. Observed trends in gene expression were consistent with involvement of all of these processes in differential recovery from SCI. We propose a model in which T cells contribute to early tissue damage, demyelination, and K_v_ channel dysregulation in SD rats following contusion SCI, but delayed tissue death limits the long-term recovery of AN rats.

### Immune response to SCI in AN rats

The immune response differed substantially between AN and SD rats. Among genes differentially expressed in the acute phase only, immune system-related genes were most highly represented. As expected, T cell and helper T cell marker genes were greater expressed in SD rats in the acute phase, when the T cell response is maximal [5, 6]. However, other T cell subsets appeared to be more numerous in AN rats. Cytotoxic T cell marker *CD8a* was enriched in AN rats in the acute phase, while T_h_2 cell marker *CXCR4* was enriched in AN rats in both acute and chronic phases. Shift from T_h_1 to T_h_2 has been associated with anti-inflammatory processes [39], so T_h_2 enrichment in AN rats may facilitate locomotion in the acute period.

The presence and functionality of T cells in AN rats remains an open topic of research. The absence of a functional thymus leads to production of fewer, less mature “T-like cells” [40]. Culture of T-like cells from AN rats yields cytotoxic T cells that recognize foreign antigens in vitro but not in vivo [41, 42]. T-like cells accumulate over time, reaching half of the normal T cell level by 8–12 months of age [43]. Rats in the present study, however, were injured at 4 months of age.

Elevated *CD8a* and *CXCR4* expression in AN rats conceivably might not reflect an overabundance of cytotoxic T cells and T_h_2 cells. *CD8a* is also expressed on NK cells [44] and CNS-infiltrating macrophages [45]. However, while *CD8a* was acutely enriched in AN rats, NK cell marker *KLRA1* was enriched in AN rats in the chronic phase only, and macrophage marker *CD68* was not differentially expressed. *CXCR4* is involved in CNS development [46], but development-related genes were predominantly enriched in SD rats. Thus, increased *CD8a* and *CXCR4* expression is consistent with greater cytotoxic T cell and T_h_2 cell infiltration in AN rats. The basis of these differences is unclear but may relate to complex T cell abnormalities in AN rats.

Highly enriched expression of macrophage-secreted cytokines IL-1a and IL-6 in AN rats may reflect compensation for the deficient T cell response. NK cells, which are actually more numerous and effective in AN rats compared to normal littermates [47], appeared to be abundant in the chronic injury site of AN rats and may similarly compensate for T cell deficiency. Conversely, chronic enrichment of mast cells in AN rats may have aided recovery, as mast cells have been found to reduce inflammation following SCI [48]. The role of mast cells in CNS injury is controversial, however, as mast cells have been reported to exacerbate brain injury in ischemic stroke [49] and intracerebral hemorrhage [50].

Macrophages, dendritic cells, granulocytes, and B cells play important roles in SCI [2, 51], but their respective marker genes did not vary between AN and SD rats. Macrophage recruitment and activation is driven by helper T cells, and prior research has found reduced macrophage infiltrate in AN rats compared to SD rats at 11 weeks post-injury [23]. We therefore performed fluorescent immunostaining for macrophage (CD68) and macrophage subtype (M1: CD16; M2: CD163) markers in the acute injury phase (1 week post-SCI) [52, 53]. We found no strain-dependent differences in the presence of any of these markers, consistent with our gene expression findings. It should be noted that histological differentiation of M1 and M2 macrophage subtypes is difficult [54] and is often done using double-staining methods [55], which were not feasible in our case owing to technical limitations.

### Cell death and tissue repair

Our findings suggest that while SD rats appear to suffer greater acute neuronal death, delayed cell death was more common in AN rats. In the acute phase, 61 % of cell death-associated genes and 79 % of neuron death-associated genes were enriched in SD rats; in the chronic phase, 57 % of cell death-associated genes were enriched in AN rats. Only 41 % of neuron death-associated genes were chronically enriched in AN rats; this discrepancy suggests that delayed cell death in AN rats may involve less loss of neurons than oligodendrocytes and other cells.

The predominant form of cell death appears to have been apoptosis rather than necrosis. Of note, necrosis-associated genes were enriched in AN rats, suggesting greater non-immune-mediated cell death. This is consistent with a past report of reduced apoptosis 24 h after traumatic brain injury in mice without functional T cells compared with immunocompetent mice [24].

### K_v_ channel dysregulation

Demyelination of intact axons uncovers K_v_ channels located in the juxtaparanodal and internodal regions [56, 57]. Exposure of these channels leads to decreased axonal excitability and creates a conduction block [58, 59]. This problem is compounded by increased K_v_ expression [58] and redistribution of K_v_ channels along axons [57, 58].

Conceivably, if T cell activity causes increased cell death and demyelination, K_v_ channel dysregulation may contribute to the transient locomotor superiority of AN rats after contusion SCI. Consistent with this hypothesis, we found that nearly all differentially expressed K_v_ channel-related genes were enriched in SD rats in both the acute and chronic phases. Notably, the K_v_ channel clustering gene *CNTN2* was greater expressed in SD rats in the acute phase only. *CNTN2* (contactin-2, also known as *TAG*-*1*) is necessary to confine K_v_ channels to the juxtaparanodal region [60]. Expression of *CNTN2* has been found to increase after SCI, and *CNTN2* dysfunction is associated with worse motor recovery and decreased axonal regrowth following injury [61]. Thus, increased expression of *CNTN2* and K_v_ channel-related genes may occur in response to greater early demyelination in SD rats. Delayed demyelination (e.g. due to oligodendrocyte death—see above) in AN rats may explain the absence of chronic differential expression of *CNTN2*.

### Other contributing factors

Despite greater acute tissue damage, SD rats may have undergone greater axonal regrowth. Neurofilament expression was strongly elevated in SD rats in the acute (FC = −3.31, Q = 0.05) and chronic (FC = −1.90, Q < 0.01) phases. Axon guidance-related genes were highlighted in GSEA and were highly enriched in SD rats. Axon guidance refers to migration of the axon growth cone, and like increased neurofilament synthesis, enrichment of axon guidance-related genes suggests increased axonal repair in SD rats. Axonal tracing studies are necessary to test this hypothesis. Local sprouting and long-distance axonal regeneration are known to occur after contusion SCI [36, 37]. However, these changes are believed to produce little if any functional improvement [38] and are therefore unlikely to play a major role here.

Edema is a prominent feature of acute SCI. Vascular injury and blood–brain barrier disruption lead to vasogenic edema, which contributes to early spinal cord dysfunction [38]. While not measured in the present study, the volume of edematous spinal cord tissue—identified on MRI as the *T*_2_-hyperintense region—has been found to be identical in AN and SD rats throughout recovery from SCI [25]. Therefore, it seems unlikely that edema is responsible for strain-dependent variation in locomotor recovery.

As previously stated, we did not assess all pathophysiological processes involved in SCI. Reactive oxygen species production, glutamate-mediated excitotoxicity, and microvascular dysfunction were less well suited to gene expression-based analysis than cell death, tissue repair, and K_v_ channel dysregulation. While the potential relationship of these unexamined processes to T cell activity is unclear, they may nevertheless contribute to the effects of T cells on functional recovery.

Differences between the genomes of AN and SD rats warrant consideration as well. The AN rat is a homozygous *rnu*/*rnu* mutant derived from a colony of hooded rats [62, 63]. To our knowledge, there is no published comparison of the AN and SD genomes. Functional SCI outcomes are known to vary between common laboratory rat strains [64, 65], raising the possibility that non-T cell-related genes may account for the locomotor difference observed in the present study. However, the albino SD rat demonstrates higher BBB locomotor scores after SCI compared to the hooded Wistar and Long-Evans rats [64]. This suggests that if background genomic inequality is present, AN rats are more likely at a disadvantage—rather than an advantage—compared to SD rats.

## Conclusions

Taken together, these findings implicate an altered immune response with relevant pro- and anti-inflammatory elements in the altered course of locomotor recovery of AN rats. Less damage by T cells—coupled with anti-inflammatory effects of T_h_2 cells (albeit of questionable functionality) and mast cells—may have led to temporary tissue preservation. The absence of superior long-term functional recovery may be due to compensatory NK cell overabundance and macrophage overproduction of inflammatory cytokines IL-1a and IL-6. We posit that T cells promote early tissue damage, demyelination, and K_v_ channel dysregulation after contusion SCI. However, compensatory features of the immune response (e.g. higher NK cell activity and increased IL-1a and IL-6 production) may cause delayed tissue death in T cell-deficient AN rats, limiting long-term recovery.

By nature, this work constitutes a broad-spectrum analysis of the role of T cell deficiency in SCI. While we have identified many promising correlations between gene expression and locomotor recovery, these relationships require extensive confirmatory studies. Several factors involved in the pathology of SCI were not investigated in this study and may contribute to the effect of T cells on functional recovery. The mechanistic basis for certain aspects of the AN immune response (particularly increased T_h_2 and cytotoxic T cell involvement) remains unclear, and may serve as a basis for subsequent experiments. Many studies—particularly systematic, quantitative immunohistochemistry—are thus required to corroborate and expand upon our findings.

Beyond helping to further elucidate the role of T cells in recovery from spinal cord trauma, our findings may inform future investigation of therapy for SCI. We have proposed that the immune consequences of T cell deficiency permit sparing of spinal cord tissue that is eventually lost in both immunodeficiency and immunocompetence. Combination therapy with T cell inhibitors and other neuroprotective treatment may allow continued survival of these vulnerable tissues and produce sustained functional recovery.

## Additional file

RNA-seq data (raw and processed files) are available in the Gene Expression Omnibus (http://www.ncbi.nlm.nih.gov/geo) under accession number GSE62760.

10.1186/s12868-015-0212-0 Complete list of identified genes, expression values, and significance testing.

## Abbreviations

AN rat: athymic nude rat
FC: fold change (AN FKPM divided by SD FKPM)
FPKM: fragments per kilobase of exon per million fragments mapped
GO: gene ontology
GSEA: gene set enrichment analysis
K_V_ channel: voltage-gated potassium channel
SCI: spinal cord injury
SD rat: Sprague–Dawley rat
